# Antimicrobial Resistance Factors of Extended-Spectrum Beta-Lactamases Producing *Escherichia coli* and *Klebsiella pneumoniae* Isolated from Cattle Farms and Raw Beef in North-West Province, South Africa

**DOI:** 10.1155/2019/4318306

**Published:** 2019-11-06

**Authors:** Kotsoana Peter Montso, Sicelo Beauty Dlamini, Ajay Kumar, Collins Njie Ateba

**Affiliations:** ^1^Department of Microbiology, Faculty of Natural and Agricultural Sciences, North-West University, Private Bag X2046, Mmabatho 2735, South Africa; ^2^Department of Microbiology, School of Bioengineering and Biosciences, Lovely Professional University, Jalandhar-Delhi G.T. Road, Phagwara, Punjab, India

## Abstract

**Background:**

Extended spectrum beta-lactamases (ESBLs) producing Enterobacteriaceae cause severe infections in humans which leads to complicated diseases. There is increasing evidence that cattle contribute to the development and spread of multidrug resistant pathogens and this raises public health concern. Despite this, data on the concurrence of ESBL producing pathogens in cattle, especially in the North-West province are rare. Therefore, the aim of the present study was to isolate, identify and characterise ESBL producing *E. coli* and *K. pneumoniae* species from cattle faeces and raw beef samples.

**Results:**

A total of 151 samples comprising 55 faeces samples and 96 raw beef samples were collected and 259 nonreplicative potential isolates of Enterobacteriaceae were obtained. One hundred and ninety-six isolates were confirmed as *E. coli* (114; 44%) and *K. pneumoniae* (82; 32%) species through amplification of *uspA* and *uidA* and *ntrA* gene fragments, respectively. Antimicrobial susceptibility test revealed that large proportions (66.7–100%) of the isolates were resistant to Amoxicillin, Aztreonam, Ceftazidime, Cefotaxime, and Piperacillin and were multidrug resistant isolates. Cluster analysis of antibiotic inhibition zone diameter data revealed close similarities between isolates from different sources or species thus suggested a link in antibiotic exposures. The isolates showing phenotypic resistance against ESBL antimicrobial susceptibility tests were screened for the presence of ESBL gene determinants. It was observed that 53.1% of the isolates harboured ESBL gene determinants. The *blaTEM*, *blaSHV* and *blaCTX-M *genes were detected in *E. coli *isolates (85.5%, 69.6%, and 58%, respectively) while *blaCTX-*M and *blaOXA* were detected in *K. pneumoniae *(40% and 42.9%, respectively). All the genetically confirmed ESBL producing *E. coli *and *K. pneumoniae *isolates were subjected to Enterobacterial Repetitive Intergenic Consensus (ERIC) PCR analysis. Fingerprinting data revealed great similarities between isolates from different areas and sources which indicates cross-contamination between cattle and beef.

**Conclusion:**

This study revealed that cattle and its associated food products, beef in particular, harbour ESBL producing pathogens. And this warrants a need to enforce hygiene measures and to develop other mitigation strategies to minimise the spread of antibiotic resistant pathogens from animals to human.

## 1. Introduction

Extended spectrum beta-lactamases (ESBL) are enzymes that can hydrolyse various *β*-lactam antibiotics and thus mediate resistance to penicillins, 3^rd^ and 4^th^ generation cephalosporins [1]. The genes encoding for those enzymes are commonly found both in the chromosomes and plasmids among the species belonging to Enterobacteriaceae family. As a result, ESBLs have emerged as a cause of resistance in Enterobacteriaceae, particularly *E. coli* and *Klebsiella* species. This phenomenon was first reported in 1980s in Europe and subsequently in USA [2]. Different antibiotic groups are applied at both therapeutic and subtherapeutic levels in the management of farm animals. Beta-lactam antibiotics are widely used in veterinary medicine precisely due to their high specificity, perfect selective toxicity, and potent killing effects [3, 4]. Thus excessive use of these antibiotics in veterinary medicine exacerbated the emergence and dissemination of genetic determinants, particularly in *E. coli* and *K. pneumoniae* species [5].

ESBL producing *E. coli* and *Klebsiella* species cause severe infections in humans even in countries with advanced public health and health care facilities [5, 6]. And most of infections are associated with cross contamination in hospital and clinic settings. Despite this, community acquired infections have also been reported in several countries worldwide [2, 7, 8]. Given that the natural hosts of *E. coli* and *Klebsiella* species are ruminants especially cattle, community acquired infections have been linked to the consumption of contaminated food particularly meat [3, 9]. ESBL producing *E. coli* and *Klebsiella* species may be transmitted to meat if standard operating procedures as well as proper hygiene practices are not implemented in the farms and abattoirs, respectively [9–12]. Despite the public health significance of ESBL producing strains globally, and the need to assess their occurrence in food producing animals [9–11, 13], only one report has been in the Eastern Cape Province of South Africa [14].

Agriculture contributes about 2.6% to the total GDPR and 19% to formal employment in the North-West province and it is of extreme importance to the inhabitants [15]. Despite the fact that the North-West province is known to be an important food basket in South Africa, with Maize and sunflower as the most important crops, the province is well known for cattle farming and it is sometimes referred to as the Texas of South Africa. The largest percentage of grazing land and cattle herds are seen in Stella and near Vryburg. In addition, a wide range of livestock farming, which includes cattle, sheep, goats, and chicken farming, is practiced in the Marico region. This kind of farming contributes a substantial percentage towards the economic growth of the area. Meat and dairy products are the main products produced, with commercial game ranching also contributing through the supply of meat.

The frequent use of antibiotics to enhance animal productivity provides opportunities for bacteria that harbour resistance determinants to be released into the environment through the discharge of faecal matter from animals, and this poses severe epidemiological implications on humans [16], given that the province is registered as the forth in terms of the prevalence HIV/AIDS in the country. In this paper, we report on the antimicrobial resistance profiles of ESBL producing *E. coli* and *Klebsiella* species isolated from faecal samples collected from cattle farms as well as meat from randomly selected supermarkets in the North-West province of South Africa as part of a larger study designed to use bacteriophages as biocontrol agents for multiple antibiotic resistant bacteria in the environment and the South African food chain.

## 2. Materials and Methods

### 2.1. Sample Collection

Sampling was done between August 2014 and May 2015. A total of 55 faeces samples were collected from 2 commercial cattle farms in Mafikeng District of the North-West province for the study. Faecal samples were collected directly from the rectum of individual animals using sterile arm-length gloves and in order to avoid duplication of sampling, the cattle were looked in their respective handling pens. In addition, 96 raw beef samples were collected from butcheries, supermarkets, and retails in 11 major cities of the four Districts of the North-West province. After collection, samples were transported on ice to the North-West University Molecular Microbiology laboratory for immediate processing. Data on antibiotic type and treatment history were collected for the purpose of understanding antibiotic exposure histories of isolates from the study population.

### 2.2. Isolation and Identification of E. coli and Klebsiella Species

Approximately, two grams (2 g) of each meat sample was washed in 5 mL of 2% (w/v) buffered peptone water (BPW) obtained from Biolab, South Africa while 2 g of faecal samples was dissolved in 5 mL of tryptic soya broth (TSB) (Merck (Pty) Ltd, South Africa). Ten-fold serial dilutions of the samples were prepared using 2% (w/v) buffered peptone water as the diluent [17]. Aliquots of 100 µL from each dilution were spread-plated on MacConkey agar (with crystal violet) (Merck (Pty) Ltd, South Africa) plates and plates were incubated aerobically at 37°C for 24 hours. After incubation, single pale, colourless, and pink colonies were sub-cultured onto MacConkey agar (with crystal violet) plates. One colony per plate was picked into a sterile tryptic soya broth and further incubated aerobically at 37°C for 24 hours for glycerol stock preparation and isolates were preserved at −80°C for future use.

### 2.3. Preliminary Identification Tests

The pure colonies were subjected to Gram-staining [18] and the oxidase test, TSI test [19] as well as the Simmons' citrate agar test to screen for characters of *E. coli *and *Klebsiella* species.

### 2.4. Phenotypic Screening of Isolates for ESBL Production

All isolates were screened for ESBL production on Brilliance ESBL Agar plates [20] obtained from Oxoid, Basingstoke, United Kingdom. In order to perform this, isolates were subcultured in Brilliance ESBL Agar and plates were incubated aerobically at 37°C for 24 hours. ESBL-producing *E. coli *and *Klebsiella* species were identified by the presence of blue or pink and green colonies, respectively. For quality control, *E. coli* (ATCC 25922) and *K. pneumoniae* (ATCC 13883) were used as a negative control. Potential *E. coli* and *Klebsiella* isolates were further subjected to the API 20E analytical assay following the manufacturer's instructions (BioMériux, France).

### 2.5. Extraction of Genomic DNA

Bacteria chromosomal DNA was extracted from all presumptive isolates using Zymo Research Genomic DNA^TM^–Tissue MiniPrep Kit following the manufacturer's instructions (Inqaba Biotechnical Industry Ltd, Pretoria, South Africa). The quality and quantity of the extracted DNA was determined using a UV–Vis Thermo Scientific™ NanoDrop Lite Spectrophotometer (model S-22, Boeco, Germany). The DNA samples were stored at −80°C for future use.

### 2.6. Molecular Identification of E. coli and Klebsiella Species by PCR Analysis

As an internal control all DNA samples were screened for bacterial 16S rRNA gene fragments using the 27F and 1492R universal oligonucleotide primer sequences [21] and were synthesised by Inqaba Biotechnical Industries, [PTY] Ltd, South Africa), and are shown in Table 1.

### 2.7. E. coli and Klebsiella pneumoniae Species Specific PCR Identification Tests

Amplification of the *uidA *and *uspA* housekeeping genes specific to *E. coli* species and* ntrA* gene fragments related to *K. pneumoniae* specific sequences was performed following previous protocols [22, 23], with minor modifications. PCR reaction mixtures were prepared as standard 25 *µ*L volumes that constituted 12.5 *µ*L of 2X DreamTaq Green Master Mix, 11 *µ*L RNase free PCR water, 0.5 *µ*L mixture of the forward and reverse primers (0.25 *µ*L of each primer) and 1 *µ*L of template DNA. All the PCR reagents were Fermentas USA products supplied by Inqaba Biotechnical Industry Ltd, Sunnyside, South Africa. Amplifications were performed using DNA thermal cycler (model-Bio-Rad C1000 Touch TM Thermal Cycler) obtained from Bio-Rad Laboratories, Inc. USA, oligonucleotide primer sequences synthesised by Inqaba Biotechnical Industries (Pty) Ltd, Pretoria, South Africa and conditions that appear in Table 1. PCR amplicons were stored at 4°C until electrophoresis.

### 2.8. Sequencing of PCR Amplicons

Bacterial 16S rRNA gene fragments were sequenced by Inqaba Biotechnical Industries (Pty) Ltd, Pretoria, South Africa and sequences were subjected to a Blast Search Tool (http://blast.ncbi.nlm.nmih.gov/Blast.cg) in order to confirm the identities of the isolates.

### 2.9. Antimicrobial Susceptibility Test

An *in-vitro* antimicrobial susceptibility test was performed on all isolates according to the Kirby-Bauer disk-diffusion method [24] in order to determine antibiotic resistant profiles and making use of antibiotic discs (Mast Diagnostics, Merseyside, UK) which were placed on inoculated Muller Hinton agar (MHA) plates. The following antibiotic impregnated discs were used: Amoxicillin (10 *µ*g), Piperacillin (100 *µ*g), Cephalothin (30 *µ*g), Cefotaxime (30 *µ*g), Ceftazidime (30 *µ*g), Cefepime (30 *µ*g), Cefoxitin (30 *µ*g), Aztreonam (30 *µ*g), and Ertapenem (10 *µ*g), and these antibiotic discs contained the CLSI approved concentrations [25]. Antibiotic growth inhibition zone diameter data were compared with standard reference values in order to classify the isolates as sensitive, intermediate resistance or resistant to a particular antibiotic [25]. In the evaluation of the results, strains displaying intermediate resistance were regarded as resistant. *E. coli* ATCC 25922 and *K. pneumoniae* ATCC 13883 strains were used as positive controls.

### 2.10. Detection of ESBL Genes in E. coli and Klebsiella Species by Multiplex PCR Analysis

All confirmed *E. coli *and *Klebsiella* isolates were screened for the presence of the *blaCTX-M*, *blaOXA*, *blaSHV*, *blaTEM*, *blaCMY*, *blaCMY-1*, and *blaCMY-2 *ESBL producing genes determinants using previously described PCR protocol [26, 27]. The primer sequences, targeted genes, amplicon sizes, and PCR conditions are listed in Table 2. The reactions were prepared in standard 25 *µ*L reaction volumes that comprised 12.5 *µ*L of a 2X DreamTaq Green Master Mix, 11 *µ*L nuclease free water, 0.25 *µ*L set of each primer, and 1 *µ*L of template DNA. All the PCR reagents were Fermentas USA products supplied by Inqaba Biotechnical Industry Ltd, Sunnyside, South Africa. The amplifications were performed using DNA thermal cycler (model-Bio-Rad C1000 Touch TM Thermal Cycler) obtained from Bio-Rad Laboratories, Inc. USA and cycling conditions indicated in Table 2. PCR amplicons were held at 4°C until resolved by electrophoresis.

### 2.11. ERIC-PCR Genetic Typing of ESBL Producing E. coli and K. pneumoniae Isolates

All ESBL producing *E. coli* and *K. pneumoniae* isolates were subjected to ERIC-PCR in order to determine the genetic similarities between the isolates. ERIC-PCR was performed using a single oligonucleotide primer ERIC2 (5′-AAGTAAGTGACTGGGGTGAGCG-3′) as previously described [28]. The ERIC fingerprints were compared and analysed for the presence, absence, and intensity of band data obtained.

### 2.12. Electrophoresis of PCR Products

PCR products were separated by electrophoresis on a 2% (w/v) agarose gel containing ethidium bromide (0.1 *µ*g/mL) (Sambrook et al., 1989). Depending on the size of the amplicons, a 1 Kb or 100 bp DNA molecular weight marker (Fermentas, USA) was included in each gel. Electrophoresis was conducted in a horizontal Pharmacia biotech equipment system (model Hoefer HE 99X, Amersham Pharmacia biotech, Sweden) for 1 hour at 80 V using 1X (v/v) TAE buffer. A ChemiDoc Imaging System (Bio-RAD ChemiDocTM MP Imaging System, UK) was used to capture the image using Gene Snap (version 6.00.22) software (GSL Biotech Chicago, USA).

### 2.13. Statistical Analysis

Statistical analysis of antibiotic resistance data was performed using the Minitab Release software (version 13.31) produced by Minitab, LLC, Pennsylvania, USA. Correlations between antibiotic resistant isolates from the various sources were determined using the percentage antibiotic resistance for each antibiotic the Pearson's product of moment and scored as significant if *p* ≤ 0.05. Furthermore, cluster analysis of isolates from the different stations was determined using bacterial growth inhibition zone diameter data obtained from antibiotic susceptibility tests on Statistica version 12 (Statsoft, US). Analysis was performed using Wards algorithm and Euclidean distances [34].

## 3. Results

### 3.1. Detection of E. coli and Klebsiella pneumoniae Isolated from Faecal and Beef Samples

**Figure 1:**
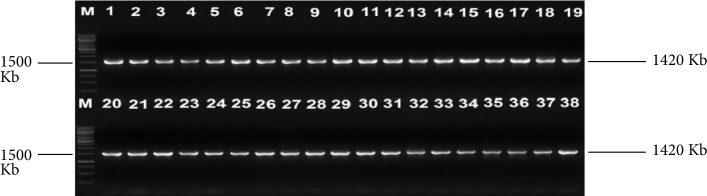
A 2% (w/v) agarose gel of 16S rRNA gene fragments amplified from *E. coli* and *Klebsiella* isolates as well as *E. coli* (ATCC 25922) and *K. pneumoniae *(ATCC 13883) control strains. Lane M = DNA marker (1 kb O'GeneRuler DNA marker), Lane 1 = *E. coli* (ATCC 25922), Lane 2 = *K. pneumoniae* (ATCC 13883), Lanes 3–19 = 16S rRNA gene fragments of *E. coli* isolates from cattle faeces and beef samples, Lanes 20–38 = 16S rRNA gene fragments of *K. pneumoniae* isolates from cattle faeces and beef samples.

**Figure 2:**
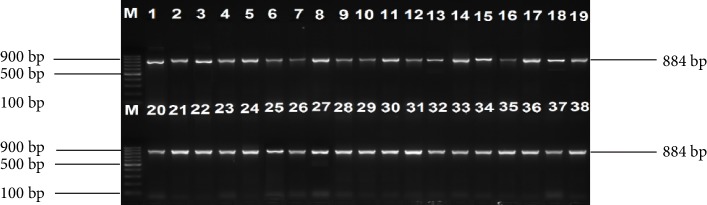
A 2% (w/v) agarose gel image showing the *uspA* gene fragments amplified from all *E. coli* isolates and *E. coli* (ATCC 25922) control strain. Lane M = 100 bp DNA Ladder, Lane 1 = *uspA* gene fragments amplified from *E. coli* (ATCC 25922) control strain, Lanes 2–38 = *uspA* gene fragments amplified from *E. coli* isolates from cattle faeces and beef samples.

**Figure 3:**
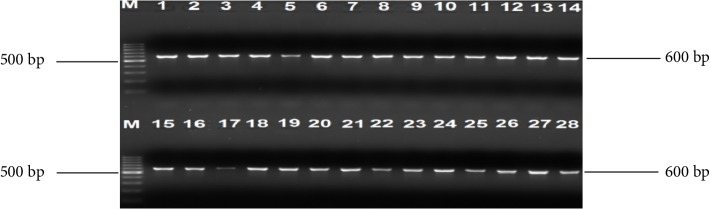
A 2% (w/v) agarose gel image showing *uidA* gene fragments amplified from all *E. coli* isolates and *E. coli *(ATCC 25922) control strain. Lane M = 100 bp DNA Ladder, Lane 1 = *uidA* gene fragments amplified from *E. coli* (ATCC 25922) control strain, Lanes 2–28 = *uidA* gene fragments amplified from *E. coli* isolates from cattle faeces and beef samples.

**Figure 4:**
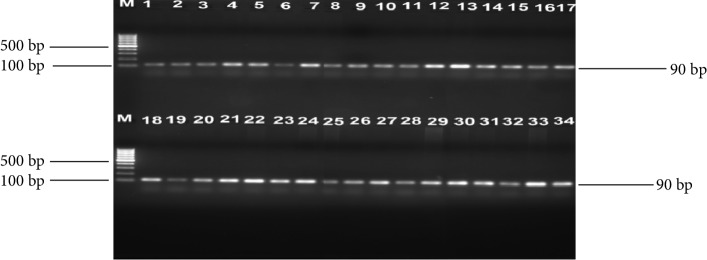
A 2% (w/v) agarose gel image showing *ntrA* gene fragments amplified from all *K. pneumoniae* isolates and *K. pneumoniae *(ATCC 13883) control strain. Lane M = 100 bp DNA Ladder, Lane 1 = *ntrA* gene fragments amplified from *K. pneumoniae* (ATCC 13883) control strain and Lanes 2–34 = *ntrA* gene fragments amplified from *K. pneumoniae* isolates from cattle faeces and beef samples.

**Figure 5:**
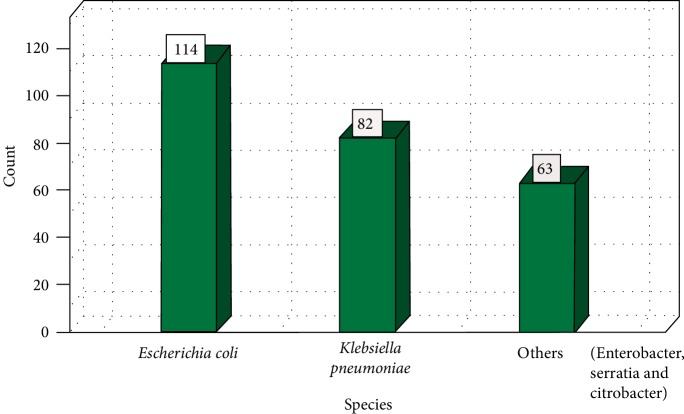
Distribution of *E. coli* and *K. pneumoniae* isolated from beef and faeces samples based on genus specific PCR analysis.

One hundred and fifty-one (55 cattle faeces and 96 raw beef) samples were collected and analysed. A total of 259 nonreplicative presumptive isolates were selected based on differences in colonial morphologies. All the isolates were Gram-negative rod shaped bacteria that were oxidase positive and hydrolysed the substrates glucose, sucrose and lactose at sample concentrations of 0.1, 1.0, and 1.0%, respectively in the TSI medium. Out of 259 isolates that were subjected to API 20E assay, 145 (56%) *E. coli* and 114 (44%) *Klebsiella* species were positively identified. Large proportions 196 (76.4%) of these isolates were confirmed as ESBL producing strains based on activity on Brilliance ESBL agar and this comprised 114 (58.2%) *E. coli* (blue colonies) and 82 (41.8%)* Klebsiella* species (green colonies). As an internal control the 16S rRNA gene fragment was successfully amplified in all the 259 isolates and Figure 1 indicates a 2% (w/v) agarose gel image of the bacterial 16S rRNA gene fragments. Large proportions 44% of these isolates were confirmed as *E. coli*, while 32% were positive for *Klebsiellapneumoniae* through amplification of *uidA* and *uspA* and *ntrA* gene fragments, respectively. Figures 2–4 indicate 2% agarose gel images of the *uidA*, *uspA* and *ntrA* gene fragments amplified in the study. A total of 63 (24.3%) isolates that were negative for *E. coli *and *Klebsiella pneumoniae* specific sequences were classified as others (Figure 5).

### 3.2. 16S rRNA Gene Sequencing

The 16S rRNA gene sequence data indicated that *E. coli *isolates possessed great (97% to 99%) similarities to *E. coli* strain O157:H6 (Accession No: CP007592.1), *E. coli *strain C15 (Accession No: CP011018.1) and *E. coli *strain SUS3EC (Accession No: KF991476.1) 16S ribosomal RNA gene, partial sequence. In addition, *K. pneumoniae *isolates possessed 95% sequence similarities to *K. pneumoniae *strain QLR-1 (Accession No: KM096433.1) and a *K. pneumoniae *strain (Accession No: HG416956.1) 16S ribosomal RNA gene, partial sequence.

### 3.3. Antibiotic Susceptibility Profiles of Isolates

A total of 196 PCR confirmed *E. coli* and *Klebsiellapneumoniae* isolates that revealed ESBL traits on Brilliance ESBL agar were subjected to antimicrobial susceptibility test in order to evaluate their resistance patterns. The number of isolates that was resistant to the different antimicrobial agents was translated into percentages in Table 3. Large proportions (85–100%) of the isolates from all the sampling sites except for those from samples from Mafikeng (54.5%) and Boshoek (66.7%) were resistant to Ampicillin. In addition, significant proportions (66.7–100%) of the isolates from Stella and Boshoek were resistant to Cefotaxime, Piperacillin, Ceftazidime, and Aztreonam. Similarly, large proportions (90%) of the isolates from Potchefstroom were resistant to Amoxicillin, Cephalothin, and Piperacillin. Despite the fact that some isolates obtained in the study displayed low levels of resistance to some of the antibiotics tested, the detection of multi-drug resistant isolates was a cause for concern since they may pose severe health complications on humans.

### 3.4. Cluster Analysis of E. coli and Klebsiella pneumoniae Species for Antibiotic Resistance Relationship

**Figure 6:**
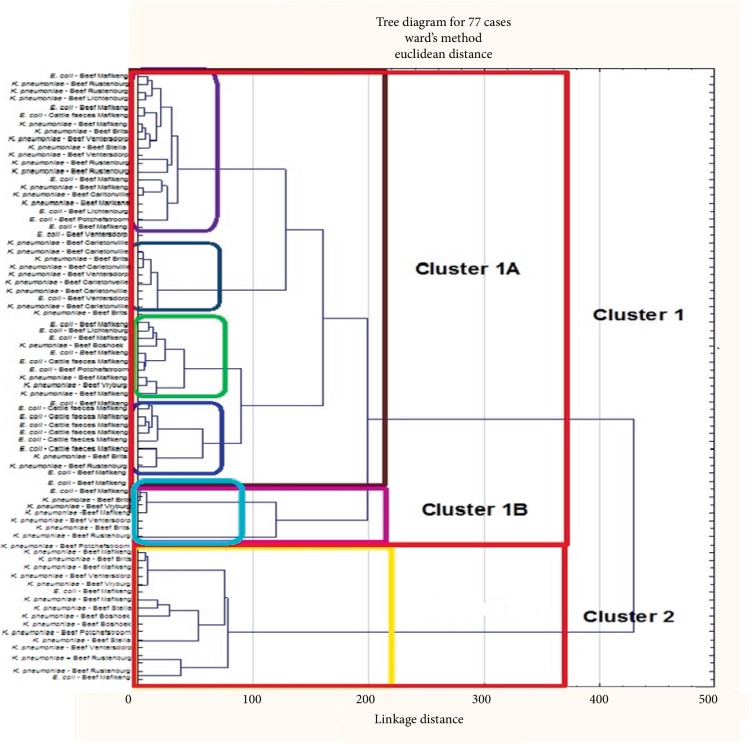
Dendrogram showing the relationship between isolates from cattle faeces and raw beef samples based on antimicrobial inhibition zone diameter data.

A total of 77 multi-drug resistant isolates that comprised 40 *E. coli *and 37 *Klebsiella pneumoniae *were randomly selected from all sampling locations and subjected to cluster analysis using their antibiotic growth inhibition zone diameter data. Random selection was due to the fact that the software can only accommodate a maximum of 77 isolates. A total of 52 isolates formed reliable cluster patterns and two main clusters (Cluster 1 and Cluster 2) were identified (Figure 6). The largest cluster (Cluster 1) contained 43 isolates and it was divided into two subclusters (Cluster 1A = 39) and (Cluster 1B = 9). The clusters were analysed for patterns of association of isolates from different sources and/or locations and data are presented in Table 4.

Subcluster 1A was considered a mixed cluster since it contained isolates from all the 11 sampling sites and large proportions (28.2% and 20.5%) of these isolates were obtained from beef and cattle faeces samples, respectively. On the contrary, subcluster 1B as well as cluster 2 did not contain any of the isolates obtained from cattle faeces in the study. In addition, a significant proportion (33.3%) of the isolates from samples collected in Mafikeng were dominant in cluster 2 as well as small proportions (11.1%) of isolates from the other six (6) sampling sites. Subcluster 1B comprised 25% and 50% of the isolates from Brits and Mafikeng, respectively. The great similarities in the antimicrobial resistant profiles of the isolates from different sampling sites clearly indicate similarities in antibiotic exposure histories.

### 3.5. Molecular Detection of ESBL Determinant Genes in *E. coli* and *K. pneumoniae* Isolates

**Figure 7:**
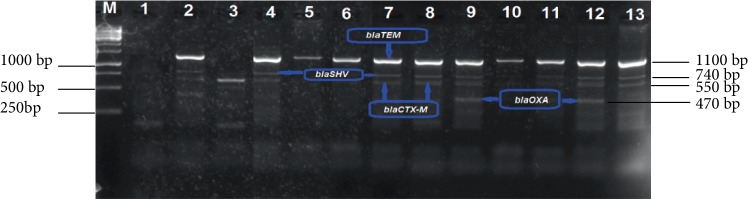
A 2% (w/v) agarose gel image showing ESBL [*bla_TEM_*(1100 bp), *bla_SHV_*(740 bp), *bla_CTX-M_* (550 bp) and *bla_OXA_* (470 bp)] gene fragments amplified from *E. coli *isolates. Lane M = 1 kb DNA marker, Lane 1 = negative control, Lanes 5, 6, and 10 = *bla*_TEM_ gene fragments, Lanes 2, 9, and 12 = *bla_TEM_*, *bla_SHV_*, *bla_CTX-M_*, and *bla_OXA_* gene fragments, Lane 3 = *bla_CTX-M_* gene fragments and Lanes 4, 7, 8, 11, and 13 = *bla_TEM_*, *bla_SHV_*, and *bla_CTX-M_* gene fragments amplified from *E. coli* isolates.

**Figure 8:**
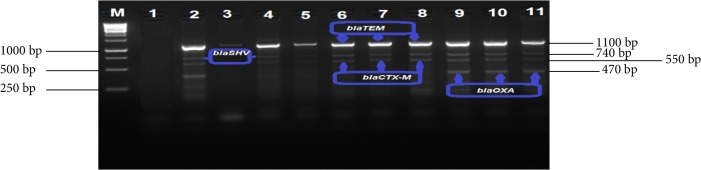
A 2% (w/v) agarose gel image showing ESBL [*bla_TEM_* (1100 bp), *bla_SHV_* (740 bp), *bla_CTX-M_* (550 bp) and *bla_OXA_* (470 bp)] gene fragments amplified from *K. pneumoniae* isolates. Lane M = 1 kb DNA Ladder (O'GeneRuler), Lane 1 = negative control, Lanes 2, 9, 10, and 11 = *bla_TEM_*, *bla_SHV_*, *bla_CTX-M _*, and *bla_OXA_* gene fragments, Lane 3 = *bla_TEM_* gene fragments, Lanes 4, 5, 6, 7, and 8 = *bla_TEM _*, *bla_SHV_* and *bla_CTX-M_* gene fragments.

All 196 isolates that comprised 114 *E. coli* and 82 *K. pneumoniae* were screened by multiplex PCR analysis for ESBL gene determinants (*blaTEM*, *blaSHV*, *blaCTX-M*, *blaOXA*, and *blaCMY-2*). The proportion of isolates that were positive for the respective genes are shown in Table 5, while Figures 7 and 8 indicate a 2% (w/v) gel image of the *blaTEM* (1100 bp), *blaSHV* (740 bp), *blaCTX-M* (550 bp) and *blaOXA* (470 bp). Large proportions 53.1% of the isolates harboured the ESBL genes targeted in the study. In addition, ESBL gene determinants were frequently detected in *E. coli* (35%) isolates than in *K. pneumoniae* (18%) (Table 5). Moreover, all ESBL genes investigated were detected among the *E. coli* isolates and this *blaTEM* gene fragment was dominant (85.5%). Despite the fact the *blaTEM* gene was detected in 64.4% isolates, only a small proportion (22.8%) of *K. pneumoniae* isolates harboured this gene.

**Figure 9:**
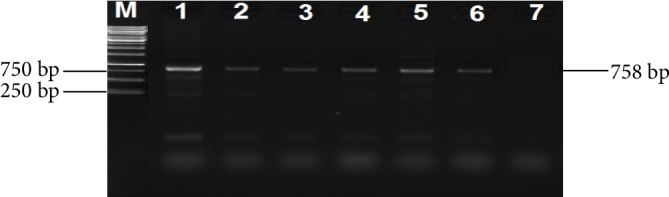
A 2% (w/v) agarose gel image showing *bla_CMY-2_* ESBL gene fragments amplified from *E. coli* isolates. Lane M = 1 kb DNA Ladder. Lanes 1–6 = *bla_CMY-2_* gene fragments amplified from *E. coli* isolates and Lane 7 = negative control.

The *blaSHV* and *blaCTX-M* were detected at proportions of 57.7% and 51.9%, respectively, among the *E. coli* and *K. pneumoniae* isolates. The *blaSHV* gene was dominant (69.6%) among *E. coli* species when compared to *K. pneumonia* (34.3%) isolates. Similarly, a larger proportion (58%) of *E. coli* isolates harboured the *blaCTX-M* gene than *K. pneumonia* (40%) isolates. As depicted in Figure 9, the *blaCMY-2* gene determinant was the least detected among all the ESBL genes targeted and was harboured by only 7 (10%) *E. coli* isolates while none of the *K. pneumoniae* isolates was positive for this gene.

### 3.6. Genotypic Typing of ESBL Producing E. coli and Klebsiella pneumoniae Isolated from Faecal and Beef Samples

**Figure 10:**
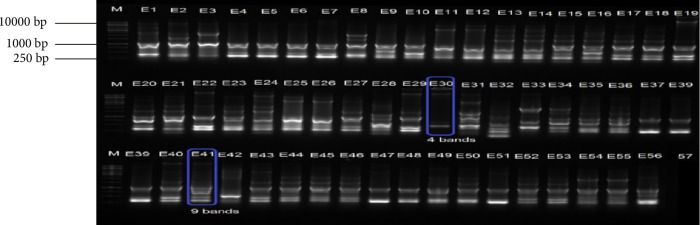
A 2% (w/v) agarose gel image depicting Enterobacterial Repetitive Intergenic Consensus (ERIC) fingerprints of representative *E. coli* isolates. Lane M = 1 kb DNA ladder, Lanes 1–32 = ERIC fingerprints of *E. coli* isolates from beef samples, Lanes 33–56 = ERIC fingerprints of *E. coli* isolates from cattle faeces and Lane 57 = Negative control.

**Figure 11:**
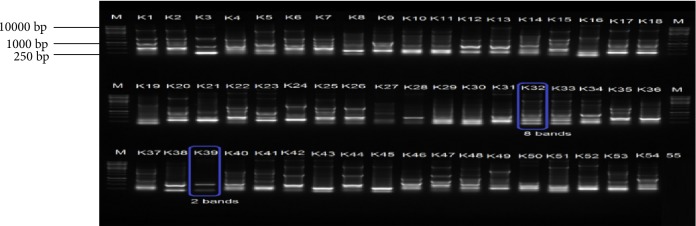
A 2% (w/v) agarose gel image depicting Enterobacterial Repetitive Intergenic Consensus (ERIC) fingerprints of representative *K. pneumoniae* isolates. Lane M = 1 kb DNA ladder, Lanes 1–54= ERIC fingerprints of *K. pneumoniae* isolates from beef samples and Lanes 55 = Negative control.

The genetic relatedness of 196 (114 *E. coli* and 82 *K. pneumoniae*) isolates was determined by subjecting them to ERIC PCR analysis. Results indicated great genetic similarities among isolates and fingerprinting patterns of *E. coli* isolates possessed 4 to 9 bands per isolate ranging between 0.25 kb and 10 kb (Figure 10). However, a large proportion of the *E. coli* isolates had fingerprints characterised by 6 bands per isolate. *K. pneumoniae*, produced genetic fingerprinting patterns that were characterised by 2–8 bands per isolate ranging from 0.25 kb to over 10 kb (Figure 11). Similarly, large proportions of the *K. pneumoniae* isolates produced six bands per isolate. The great genetic relatedness among *K. pneumoniae* and *E. coli* isolates detected in samples obtained from different sample sites coupled with the fact that they were recovered from both cattle and raw beef samples indicates some form of cross contamination in the food chain particularly in abattoirs, but does not exclude contamination during handling and packaging of meat. This also indicates the need to improve farm management practices in the area.

## 4. Discussions

Food-producing animals are known to be reservoirs for ESBL-producing strains, especially* E. coli *[9, 14, 29, 30]. Animals colonised with ESBL producing strains have been reported to serve as potential sources of *E. coli* and *Klebsiella pneumoniae* infections in humans particularly in rural communities [31] and this was a course for concern. The primary objective of the present study was to isolate and identify *E. coli* and *Klebsiella pneumoniae* isolates from cattle faeces and beef samples. In the present *E. coli *and *Klebsiella pneumoniae* species were successfully isolated and confirmed using biochemical tests, genus specific PCR analysis and 16S rRNA gene sequencing. The use of *uidA* and *uspA* and *ntrA* housekeeping genes to confirm the identities of *E. coli* and *Klebsiella pneumoniae* species, respectively have been applied in other studies [22, 23]. In our study, *E. coli* was dominant among isolates recovered from cattle faeces and raw beef samples when compared to *Klebsiella pneumoniae*. Similar observations have been reported [11, 12, 23] and these findings are in agreement with the generalisation that *E. coli* are highly prevalent in the gastrointestinal track of ruminants when compared to other members of the family Enterobacteriaceae [23]. This also explains why *E. coli* species are approved for use in contamination source tracking investigations.

Another objective of the study was to determine the proportion of *E. coli* and *Klebsiella pneumoniae* isolates that possessed ESBL resistant phenotypes as well as their resistant determinants. This was motivated by the fact that ESBL-producing *E. coli* and *Klebsiella pneumoniae* have been frequently found to produce extended-spectrum *β*-lactamases (ESBLs) and thus making them resistant to cephalosporin antibiotics, as well as a number of other classes of antibiotics [6]. In addition, infections caused by ESBL-producing pathogens are problematic due to the potential of harbouring coresistant determinants to other antimicrobial agents hence present severe challenge to public health practitioners resulting from limited antibiotic treatment options. In general, a large proportion 196 (76.4%) of these isolates tested in the study produced phenotypic ESBL activities on Brilliance ESBL agar. These results are in accordance with previous findings [10, 11, 14, 32, 33]. Although the results from these studies varied significantly, it has also been observed that the occurrence of pathogenic *E. coli* strains was higher in cattle faeces than in beef samples [17]. This was not surprising since *E. coli* strains are known occur as a normal flora of ruminants, especially cattle [12]. However, the most susceptible host in a given area cannot be assumed without thorough analysis since data from our previous findings revealed that pigs rather than cattle are the principal host for *E. coli* O157 strains in the North-West province of South Africa [17, 34].

Antibiotic resistance is currently a very serious problem that has received great attention from the larger scientific community due to its impact on both hospital as well as community settings [35]. This, therefore, implies that rapid detection of resistant determinants in bacteria within diagnostic laboratories and knowledge of the antibiotic resistant profiles of circulating strains is very essential for the judicious recognition of the impact of these organisms to humans in a given geographical location. Within the family Enterobacteriaceae, the production of extended spectrum beta-lactamases is currently on the increase particularly among *E. coli* and *K. pneumoniae* species [35, 36], and these enzymes mediate in cellular processes that impede on treatment of infections caused by these pathogens [35, 37].

In the present study, large proportions (54.5–100%) of the isolates were resistant to Amoxicillin. In addition, 33.3–100% of the isolates were also resistant to Piperacillin while 14.3–100% were resistant to Cefotaxime. Similar observations had been reported [14, 38]. Furthermore, significantly larger proportions (16.7–90% and 16.7–66.7%) were frequently resistant to Cephalothin and Ceftazidime, respectively. Isolates from Brits and Stella were most often resistant to these drugs when compared to those from the other sampling sites. Previous findings as well as antimicrobial usage surveillance data in the study area revealed that the frequent utilization of beta-lactam antibiotics in the treatment of bacterial infections continue to be the prominent cause of high levels of beta-lactam resistance among Gram-negative bacteria [6]. In addition, the potential of ESBL-producing organisms to resistant destruction when exposed to beta-lactam antibiotics is also enhanced by frequent mutations that occur in the gene sequences encoding for beta-lactamases [6, 7]. This could account for the high levels of resistance observed against Amoxicillin since this antimicrobial agent is extensively used as the preferred drug in the treatment of bacterial infections in both veterinary and human medicine [14]. Cluster analysis of antibiotic growth inhibition zone diameter data revealed that subcluster 1A was a mixed cluster that contained isolates from all the different sample sites. These data indicate that the antibiotypes of isolates within subcluster 1A were similar and this might be due to similar antimicrobial exposure histories. Changes in the antimicrobial phenotypes may be associated with environmental factors or the acquisition of plasmids. However, the utilization of an antibiotic phenotypic typing method in which isolates are clustered based on raw antibiotic growth inhibition diameter data was efficient in the typing and nosocomial infection surveillance of Methicillin Resistant *Staphylococcus aureus* [39]. The natural hosts of *E. coli* and *K. pneumoniae* species are ruminant animals especially cattle indicating these antimicrobial resistance pathogens can easily contaminate food products and be transmitted to humans. Therefore, an investigation of the ESBL profiles among *E. coli* and *K. pneumoniae* strains in animals is of paramount importance, and data generated may assist in limiting cross-contamination [17].

A further objective of the study was to screen *E. coli *and *K. pneumoniae *isolates for ESBL antibiotic resistance gene determinants. Despite the fact that ESBL resistance genes have most frequently been detected among *E. coli* and *K. pneumoniae* isolated from humans, clinical care and hospital facilities, a number of studies have also reported that ESBL resistance genes are harboured by *E. coli* and *K. pneumoniae* strains from food-producing animals, meat products as well as vegetables [9, 11, 14, 38, 40–44]. In most studies, ESBL genes (*blaCXT-M*, *blaTEM*, *blaSHV, blaOXA* and *blaCMY*) were the most frequently detected resistant determinants in *E. coli*, *K. pneumoniae* and *Salmonella* species [6, 14, 37]. ESBL isolates that possess these resistant determinants particularly *blaCXT-M* may pose severe public health complications to humans if consumption in contaminated food products [11, 14, 40–44].

In the present study, large proportions (53.1%) of the isolates possessed ESBL genes and the *blaCTX-M*, *blaSHV* and *blaTEM* genes most frequently detected in *E. coli *than in *K. pneumoniae* isolates. These results are in accordance with the findings of a previous study conducted in the Eastern Cape Province, South Africa as well as in India [14, 41–44]. Of great concern ESBL gene determinants detected in the present study was higher than those of a similar study previously conducted in Nigeria [38]. It is hereby suggested that the proportion of ESBL resistant gene determinants reported among bacteria isolates may be dependent on differences in geographical locations, sensitivity of the methods used to either isolate bacteria or detect resistance genes as well as the source and nature of specimens analysed.

The last objective of this study was to determine the genetic relatedness of ESBL-producing *E. coli* and *K. pneumoniae* isolates using ERIC PCR analysis. ERIC PCR analysis was usually employed to amplify various regions of DNA flanked by conserved sequences in order to generate isolate specific genetic fingerprints [28]. ERIC typing results revealed that isolates from different sources and/or locations shared similar genetic fingerprinting patterns and in particular those from cattle faeces and beef samples. The great similarities in genetic fingerprints indicate that the isolates may have originated from a common ancestral strain. ERIC-PCR band patterns of *K. pneumoniae* isolates from beef samples were very similar despite the differences in their sampling stations and this was in accordance with a previous report [45]. The findings of several studies have revealed that ERIC fingerprinting is a reliable tool for discriminating among isolates from different sources and hence considered to be a powerful tool for surveillance and control of antibiotic resistant bacteria [28, 46, 47]; these data indicate the need to improve on farm management techniques as well as standard operational procedures in abattoirs. In addition, contamination of raw beef with these multi-drug resistant pathogens may have also occurred at sale points and this, therefore, amplifies the need to enforce proper hygiene practices in supermarkets.

## 5. Conclusion

To the best of our knowledge, this is the first study to report the occurrence of ESBL producing *E. coli* and *K. pneumoniae* in cattle faeces and their associate food products (beef) in the study area. Large proportions (66.7–100%) of multidrug resistant isolates were present in the samples and these isolated clustered together based on their antibiotic inhibition zone diameter data suggesting a very close link in antibiotic exposure histories. Large proportions of these *E. coli* and *K. pneumoniae* isolates displayed ESBL phenotypic resistance traits, while 53.1% harboured ESBL gene determinants. The *blaTEM*, *blaSHV* and *blaCTX-M *genes were detected in *E. coli *isolates (85.5%, 69.6%, and 58%, respectively) while *blaCTX-*M and *blaOXA* were detected in *K. pneumoniae *(40% and 42.9%, respectively). In conclusion these findings indicate that, tracking and monitoring the spread of ESBL producing strains in food producing animals and beef at farms and sales points are urgently needed to improve public health in South Africa.

## 6. Limitations and Suggestion

There are several members of Enterobacteriaceae living in the intestines of animals and isolated from their faeces. However, in the study focus was directed at isolating *E. coli* and *Klebsiella pneumoniae* and this was a limitation for the study. Another limitation of the study was that cattle faeces were collected from one district (Mafikeng), whereas beef samples were collected from all the four districts of the North-West province. Thus, the data obtained from cattle faeces might not completely reflect the actual prevalence of ESBL producing organisms in cattle. Despite this, it is suggested that further studies should be carried out to determine the linkage of ESBL producers in animals and human and to address factors that contributes to the successful dissemination of ESBL producing strains to human.

## Figures and Tables

**Table 1:** Oligonucleotide primer sequences used for amplification of 16S rRNA universal, *uspA*, *uidA*, and *ntrA* genes and PCR cycling conditions used.

Primers	Sequence (5′–3′)	Targeted gene	Amplicon size (bp)	PCR conditions and cycles	Reference
27F	AGAGTTTGATCATGGCTCAG	*16S rRNA*	1420	1 cycle of 3 minutes at 94°C, 25 cycles of 1 minute at 94°C, 1 minute at 55°C, 2 minutes at 72°C; 1 cycle of 10 minutes at 72°C	[21]
1492R	GGTACCTTGTTACGACTT				
*uspAF*	CCGATACGCTGCCAATCAGT	*uspA*	884	1 cycle of 5 minutes at 95°C, 30 cycles of 30 seconds at 94°C, 30 seconds at 56°C, 30 seconds at 72°C; 1 cycle of 5 minutes at 72°C	[22]
*uspAR*	ACGCAGACCGTAGGCCAGAT				
*uidAF*	CTGGTATCAGCGCGAAGTCT	*uidA*	556	1 cycle of 10 minutes at 95°C, 35 cycles of 45 seconds at 95°C, 30 seconds at 59°C, 1 minute 30 seconds at 72°C; 1 cycle of 10 minutes at 72°C	[23]
*uidAR*	AGCGGGTAGATATCACACTC				
*ntrA*	CATCTCGATCTGCTGGCCAA	*ntrA*	90	1 cycle of 10 minutes at 95°C, 35 cycles of 45 seconds at 95°C, 30 seconds at 55°C, 1 minute 30 seconds at 72°C; 1 cycle of 10 minutes at 72°C	
*ntrA*	GCGCGGATCCAGCGATTGGA				

**Table 2:** Oligonucleotide primer sequences used for detection of ESBL genes and PCR cycling conditions used.

Primer	Primer sequence (5′–3′)	Target gene	Amplicons size (bp)	PCR conditions and cycles	Reference
*blaTEMF*	AAACGCTGGTGAAAGTA	*blaTEM*	822	1 cycle of 5 minutes at 94°C, 35 cycles of 30 seconds at 94°C, 1 minute at 45°C, 1 minute at 72°C; 1 cycle of 10 minutes at 72°C	[27]
*blaTEMR*	AGCGATCTGTCTAT				
*blaSHVF*	ATGCGTTATATTCGCCTGTG	*blaSHV*	753		
*blaSHVR*	TGCTTTGTTATTCGGGCCAA				
*blaCTX-MF*	CGCTTTGCGATGTGCAG	*blaCTX-M*	550		
*blaCTX-MR*	ACCGCGATATCGTTGGT				
*blaOXAF*	ATATCTCTACTGTTGCATCTCC	*blaOXA*	619		
*blaOXAR*	AAACCCTTCAAACCATCC				
*blaCMY-1F*	GTGGTGGATGCCAGCATCC	*blaCMY-1*	915	1 cycle of 3 minutes at 94°C, 25 cycles of 1 minute at 94°C, 1 minute at 58°C, 1 minute at 72°C; 1 cycle of 10 minutes at 72°C	[26]
*blaCMY-1R*	GGTCGAGCCGGTCTTGTTGAA				
*blaCMY-2F*	GCACTTAGCCACCTATACGGCAG	*blaCMY-2*	758		
*blaCMY-2R*	GCTTTTCAAGAATGCGCCAGG				

**Table 3:** Percentage antibiotic resistance pattern of the isolates from cattle faeces and beef samples.

Area	Antibiotics resistance (%)								
	FOX	CTX	KF	ETP	CPM	PRL	CAZ	ATM	A
Boshoek	33.3	100	66.7	33.3	33.3	66.7	66.7	66.7	66.7
Brits	16.7	50	16.7	33.3	33.3	33.3	16.7	16.7	100
Carletonville	16.7	66.7	33.3	33.3	33.3	50	16.7	50	100
Lichtenburg	28.6	14.3	57.1	14.3	14.3	42.9	28.6	14.3	85.7
Mafikeng	12.1	43.4	30.3	17.2	15.2	37.4	17.2	23.2	54.5
Marikana	11.1	55.6	66.7	33.3	55.6	88.9	33.3	44.4	100
Potchefstroom	10	50	90	30	30	90	40	50	90
Rustenburg	28	52	60	28	32	60	28	36	96
Stella	33.3	100	33.3	33.3	33.3	100	66.7	66.7	100
Ventersdorp	14.3	42.9	42.9	14.3	28.6	57.1	14.3	42.9	100
Vryburg	10	70	65	15	35	70	30	45	85

FOX = Cefoxitin, CTX = Cefotaxime, KF = Cephalothin, ETP = Ertapenem, CPM = Cefepime, PRL = Piperacillin, CAZ = Ceftazidime, ATM = Aztreonam, A = Amoxicillin.

**Table 4:** Percentage representation of *E. coli *and *K. pneumoniae* isolated from various areas and/or sources within different clusters.

Sampling area	Source	Cluster 1A *N* = 39	Cluster 1B *N* = 4	Cluster 2A *N* = 9
Brits	Beef	2 (5.1%)	1 (25%)	1 (11.1%)
Boshoek	Beef	1 (2.6%)	0 (0%)	1 (11.1%)
Carletonville	Beef	5 (12.8%)	0 (0%)	0 (0%)
Lichtenburg	Beef	2 (5.1%)	0 (0%)	0 (0%)
Mafikeng	Beef	11 (28.2%)	2 (50%)	3 (33.3%)
	Cattle faeces	8 (20.5%)	0 (0%)	0 (0%)
Marikana	Beef	0 (0%)	0 (0%)	0 (0%)
Potchefstroom	Beef	2 (2.6%)	0 (0%)	1 (11.1%)
Rustenburg	Beef	4 (10.3%)	1 (25%)	1 (11.1%)
Stella	Beef	1 (2.6%)	0 (0%)	1 (11.1%)
Ventersdorp	Beef	2 (2.3%)	0 (0%)	1 (11.1%)
Vryburg	Beef	1 (2.3%)	0 (0%)	0 (0%)

**Table 5:** Proportion of ESBLs genes detected from isolates obtained from cattle faeces and raw beef samples.

Bacteria species	No of isolates positive for ESBL activity	No. of isolates positive for ESBL associated genes					
		*bla_TEM_*	*bla_SHV_*	*bla_CTX-M_*	*bla_OXA_*	*bla_CMY-1_*	*bla_CMY-2_*
*E. coli*	69 (66.3%)	59 (85.5%)	48 (69.6%)	40 (58%)	20 (29%)	0 (0%)	7 (10.1%)
*K. pneumoniae*	35 (33.7%)	8 (22.9%)	12 (34.3%)	14 (40%)	15 (42.9%)	0 (0%)	0 (0%)

Total	104 (100%)	67 (64.4%)	60 (57.7%)	54 (51.9%)	35 (33.7%)	0 (0%)	7 (6.7%)

## Data Availability

No access to data on the antimicrobials used in the investigated farms; unavailability or scarcity of data from meat wholesale suppliers and the Department of Agriculture, Forestry and Fisheries of South Africa.

## Supplementary Materials

Supplementary Materials Figure S1: a flow chart illustrating sample collection and bacterial characterization.
