# Supplementary figures and images for: Antimicrobial Resistance Factors of Extended-Spectrum Beta-Lactamases Producing Escherichia coli and Klebsiella pneumoniae Isolated from Cattle Farms and Raw Beef in North-West Province, South Africa
Source: Biomed Res Int. 2019 Nov 6;2019:4318306. doi: 10.1155/2019/4318306 (PMC6935440; doi:10.1155/2019/4318306)

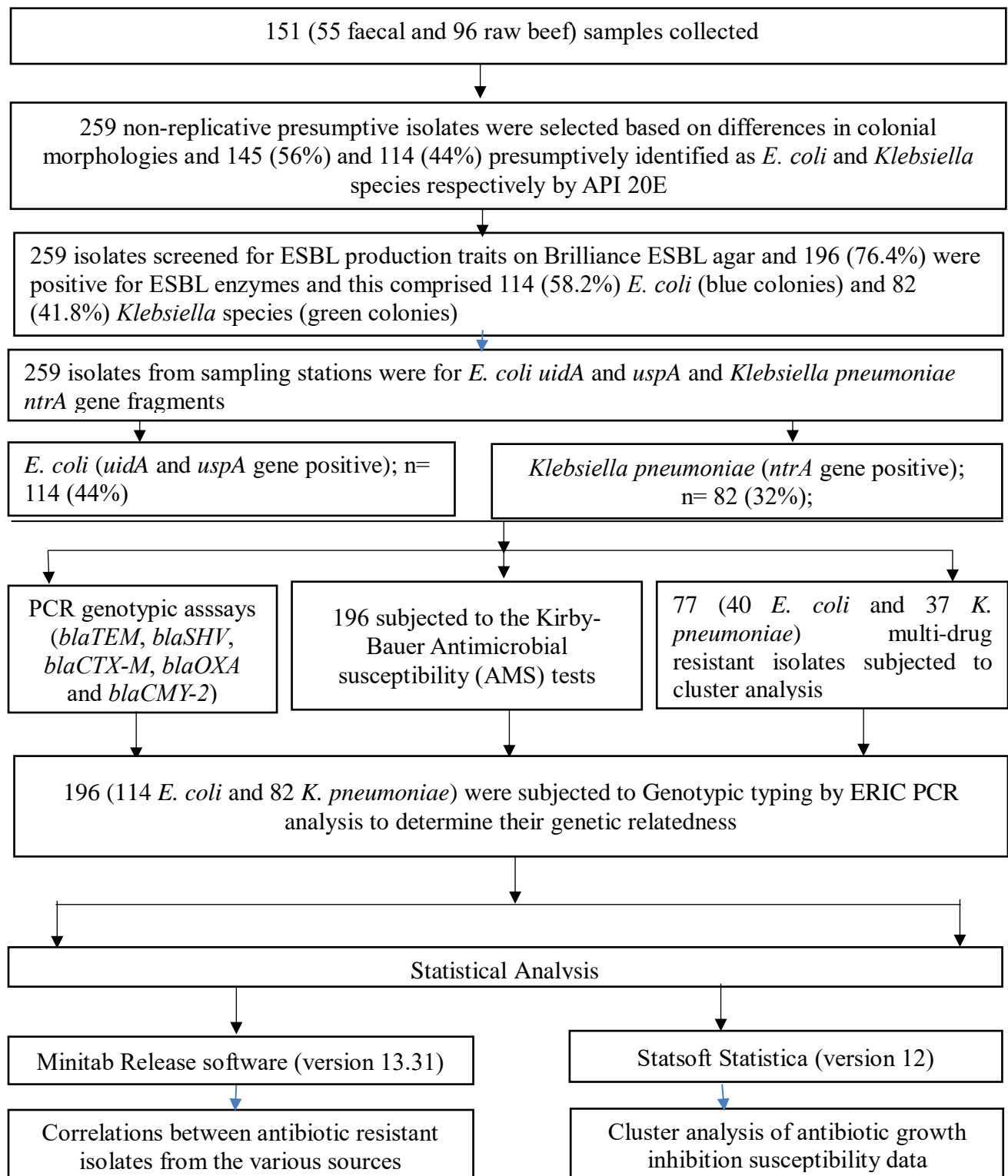

**Figure S1:** A flow chart illustrating sample collection and bacterial characterization.

Supplement: Supplementary Materials — Figure S1: a flow chart illustrating sample collection and bacterial characterization. [file 4318306.f1.pdf]
